# COVID-19 RELATED MEDICAL CARE DELAYS IN UNDERSERVED AFRICAN AMERICAN OLDER ADULTS WITH COMORBIDITIES

**DOI:** 10.1093/geroni/igac059.2989

**Published:** 2022-12-20

**Authors:** Edward Adinkrah, Sharon Cobb, Lucy Kibe, Roberto Vargas, Mohsen Bazargan

**Affiliations:** Charles R. Drew University of Medicine and Science, Los Angeles, California, United States; Charles R. Drew University of Medicine and Science, Los Angeles, California, United States; Charles R. Drew University of Medicine and Science, Los Angeles, California, United States; Charles R. Drew University of Medicine and Science, Los Angeles, California, United States; Charles R. Drew University of Medicine and Science, Los Angeles, California, United States

## Abstract

Health seeking behaviors have been negatively impacted during the COVID-19 pandemic particularly among minority older adults. There is a paucity of data concerning African American older adults who delayed, reduced, or stopped visits to primary and specialty providers. To outline the magnitude of this health disparity, this study examines the patterns and correlates of seeking preventive and specialized healthcare within this population. One-hundred and fifty (150) underserved African Americans older adults took part in this health advisor-led cross-sectional study. They completed a survey that inquired about seeking medical, dental, emergency room, and/or specialty care. We employed descriptive analyses to assess patterns of healthcare and multinomial logistic regression to document correlates of healthcare utilization. Almost one-third of participants (32%) delayed or did not get at least one type of care because of COVID-19. Dental care was the most frequent healthcare service that was missed (23%), followed by primary care (10%) and specialty care (7%). A higher level of depressive symptoms, lower level of educational attainment, and higher number of chronic conditions were associated with a higher number of delayed or cancelled medical care. Almost one-third of participants had never used any type of telehealth. Additionally, access and use of telehealth had no impact on healthcare utilization. One-year post pandemic, while vaccines are widely available, COVID-19 is disproportionately worsening healthcare disparities that exist even among African American older adults in underserved and under-resourced communities with access to healthcare. Interventional studies are urgently needed.

